# Hypothermia after Cardiac Arrest in Large Animals (HACA-LA): a randomized controlled experimental study

**DOI:** 10.1186/s40635-025-00815-y

**Published:** 2025-10-22

**Authors:** Olof Persson, Anna Valerianova, Leos Tejkl, Jan Bělohlávek, Tobias Cronberg, Niklas Nielsen, Attila Frigyesi, Susann Ullén, Wolfgang Weihs, Alexandra-Maria Stommel, Kaj Blennow, Henrik Zetterberg, Sandra Högler, Elisabet Englund, Mikuláš Mlček, Hans Friberg

**Affiliations:** 1 Department of Clinical Sciences, Anaesthesiology and Intensive Care, Lund University, 22184 Lund, Sweden; 2 Department of Intensive and Perioperative Care, Skåne University Hospital, Lund, Sweden; 3 Third Department of Internal Medicine, General University Hospital, Charles University, Prague, Czech Republic; 4 Institute of Physiology, First Faculty of Medicine, Charles University, Prague, Czech Republic; 5 Second Department of Medicine–Department of Cardiovascular Medicine, First Faculty of Medicine, Charles University and General University Hospital, Prague, Czech Republic; 6 Department of Clinical Sciences, Neurology, Lund University, Lund, Sweden; 7 Department of Neurology, Skåne University Hospital, Lund, Sweden; 8 Skåne University Hospital Lund, Lund University, and Clinical Studies Sweden—Forum South, Skåne University Hospital, Lund, Sweden; 9 Department of Emergency Medicine, Medical University of Vienna, Vienna, Austria; 10 Institute of Neuroscience and Physiology, Department of Psychiatry and Neurochemistry, The Sahlgrenska Academy at the University of Gothenburg, Gothenburg, Sweden; 11 Clinical Neurochemistry Lab, Sahlgrenska University Hospital, Mölndal, Sweden; 12 Paris Brain Institute, ICM, Pitié-Salpêtrière Hospital, Sorbonne University, Paris, France; 13 Neurodegenerative Disorder Research Center, Division of Life Sciences and Medicine, and Department of Neurology, Institute On Aging and Brain Disorders, University of Science and Technology of China and First Affiliated Hospital of USTC, Hefei, People’s Republic of China; 14 Department of Pathology and Laboratory Medicine, University of Wisconsin School of Medicine and Public Health, Madison, WI USA; 15 Wisconsin Alzheimer’s Disease Research Center, University of Wisconsin School of Medicine and Public Health, University of Wisconsin-Madison, Madison, WI USA; 16 Department of Neurodegenerative Disease, UCL Institute of Neurology, Queen Square, London, UK; 17 UK Dementia Research Institute at UCL, London, UK; 18 Hong Kong Center for Neurodegenerative Diseases, InnoHK, Hong Kong, China; 19 Centre for Brain Research, Indian Institute of Science, Bangalore, India; 20 Department of Biological Sciences and Pathobiology, University of Veterinary Medicine Vienna, Vienna, Austria; 21 Department of Clinical Sciences, Pathology, Lund University, Lund, Sweden; 22 Department of Genetics, Pathology and Molecular Diagnostics, Skåne University Hospital, Lund, Sweden; 23 Department of Intensive and Perioperative Care, Skåne University Hospital, Malmö, Sweden

**Keywords:** Cardiac arrest, Hypothermia, Temperature control, Neuronal damage, Functional outcome, Neurofilament light chain (NfL), Swine

## Abstract

**Background:**

Induced hypothermia after cardiac arrest is neuroprotective in several animal models of cardiac arrest, but few high-quality studies have been conducted in larger animals. Recent clinical trials have questioned the beneficial effects of post-ischemic hypothermia. This study investigated whether immediate cooling or a 2-h delay in cooling to 33 °C after cardiac arrest was neuroprotective compared to controlled normothermia in large animals.

**Methods:**

Young adult female swine were anesthetized and kept at normothermia (38 °C). All animals were subject to 10 min of cardiac arrest by ventricular fibrillation, followed by 4 min of cardiopulmonary resuscitation, before the first countershock. At 10 min of return of spontaneous circulation (ROSC), animals were included and randomized to receive immediate hypothermia (33 °C), 2-h delayed hypothermia (33 °C), or normothermia for 30 h, including both cooling and rewarming time. Animals were extubated and assessed for 7 days. The primary outcome was brain histopathology using a modified Histology Damage Score. Secondary outcomes were neurocognitive testing, neurologic deficit score, and biomarkers of brain injury.

**Results:**

Among 42 animals, 33 were included; 11 in each arm, 23 survived until day 7. The modified Histology Damage Score was not significantly different between groups (*p* = 0.29). Neither neurocognitive testing nor neurologic deficit scores showed significant differences between the groups (*p* = 0.11 and *p* = 0.67, respectively). Neurofilament light chain (NfL) levels were significantly lower in the immediate hypothermia group at 48 h and on day 7 compared to the normothermia group (*p* = 0.0087, *p* = 0.012), but not in the delayed hypothermia group (*p* = 0.075, *p* = 0.33).

**Conclusion:**

Our experimental model in large swine showed no neuropathological or functional protective effect of induced hypothermia after cardiac arrest, but NfL levels were lower in animals receiving immediately induced hypothermia, suggesting mitigation of neuronal injury.

*Trial registry*: Preclinicaltrials.eu (PCTE0000272), published 2021-11-03.

**Supplementary Information:**

The online version contains supplementary material available at 10.1186/s40635-025-00815-y.

## Introduction

Out-of-hospital cardiac arrest (OHCA) is a major health problem with an annual incidence of 67–170 per 100,000 inhabitants, and less than 10% survive [1]. Due to its high metabolic rate, the brain is particularly sensitive to ischemia [2]. Cardiac arrest causes an instant as well as a delayed type of brain cell death, i.e., neuronal pyknosis [3], and selective eosinophilic neuronal death (SEND) as a result of the reperfusion injury [4]. The latter pathology allows for a potential therapeutic window in post-resuscitation care [5]. Induced hypothermia decreases cerebral metabolism by 5–10% per 1 °C and mitigates several pathological processes [6, 7].

Recent reviews of induced hypothermia post-cardiac arrest in the experimental setting show a neuroprotective effect, but few studies have been conducted in large animals with gyrencephalic brains [8, 9]. Early clinical trials of post-ischemic hypothermia showed positive results [10, 11], but the beneficial effect could not be repeated in larger trials [12, 13]. An updated systematic review and meta-analysis showed no benefit of induced hypothermia after cardiac arrest [14], while another review suggests that hypothermia may be beneficial [15]. European intensive care guidelines [16] no longer recommend induced hypothermia, but controversy remains [17, 18]. While *intra-ischemic* cooling has demonstrated neuroprotective effects in experimental models [19], case reports [20], and clinical practice [21], the role and optimal timing of *post-ischemic* hypothermia in cardiac arrest remains insufficiently explored. An early study suggested a narrow therapeutic window of 15 min in dogs [22], while a recent review, primarily based on rodent models, support a window of more than 6 h [8]. The largest clinical trial to date had a median time to 34 °C of more than 5 h after cardiac arrest [13]. A return to the experimental setting is essential to investigate the potential therapeutic window of post-ischemic hypothermia in large animals.

We aimed to test the following hypotheses in large swine: (a) Mild hypothermia, induced immediately after return of spontaneous circulation (ROSC), is neuroprotective compared to normothermia, and (b) Mild hypothermia, induced with a two-hour delay after ROSC, is neuroprotective compared to normothermia.

## Methods

The Institutional Animal Expert Committee at the First Faculty of Medicine, Charles University, approved the HACA-LA study protocol. Experiments were conducted at the Experimental Laboratory, Institute of Physiology, First Faculty of Medicine, Charles University, Prague, Czech Republic, in accordance with Act No. 246/1992 Coll., on the protection of animals against cruelty and EU Directive 2010/63/EU**.** The experiments were conducted and reported in accordance with the ARRIVE and Utstein guidelines [23, 24].

Details of the methods, including: sample size calculation, acclimatization, preparation, cardiac arrest, resuscitation, monitoring, inclusion and exclusion criteria, randomization, and criteria for preemptive euthanasia, are described in a protocol paper [25].

### Experimental animals

Young adult female swine (*Sus scrofa domestica*, Large White x Landrace crossbreed), weighing 50–77 kg, were used. The animals had free access to food and water and were kept in separate boxes.

### Cardiac arrest

Animals in general anesthesia were kept at baseline parameters, including normothermia (38 ± 0.2 °C), for fifteen minutes before cardiac arrest [25]. Ventricular fibrillation (VF) was induced with rapid pacing through a pacing catheter in the right ventricle. All animals were subject to ten minutes of untreated VF, followed by four minutes of mechanical chest compressions with bag-valve ventilation, before the first countershock, details of resuscitation are described in our protocol paper [25]. Stable ROSC was defined as ten minutes of sustained systolic blood pressure > 60 mmHg; vasopressors and inotropes were allowed.

### Interventions

At stable ROSC, animals were randomized to: Normothermia (NT) group, Early hypothermia (EH) group, or Delayed hypothermia (DH) group. All animals were anesthetized, paralyzed, and kept on a ventilator during the intervention [25].

The EH group received immediate cooling via a rapid infusion (10 min) of cold (4 °C) Ringer’s Acetate (30 ml/kg) and simultaneous initiation of the endovascular cooling device (Thermoguard XP™, Zoll Medical, CA, USA), set at maximal rate, targeting 33 ± 0.2 °C. The DH group received temperature control (38 ± 0.2 °C) for two hours before initiation of the same cooling regimen (cold infusion and endovascular cooling), targeting 33 ± 0.2 °C. The NT group received an identical volume of Ringer’s Acetate at room temperature and was maintained at 38 ± 0.2 °C during the intervention. A temperature catheter was inserted into the bladder for temperature monitoring. The intervention period was thirty hours, including cooling and controlled rewarming (0.5 °C/h) [25].

### Outcomes

After the intervention period, animals were extubated and assessed for seven days.

The primary outcome was a modified Histology damage score (mHDS) at the end of the experiment [25]. Secondary outcomes included neurocognitive testing and neurologic deficit score at the end of the experiment, as well as plasma levels of biomarkers of brain injury after the intervention [25].

On day 7, the animals were euthanized with potassium chloride (2 mEq/kg) in deep general anesthesia. The brains were immersed in a 4% formaldehyde solution and embedded in paraffin. The paraffin blocks were cut into 4-µm sections and stained with hematoxylin and eosin. Two independent pathologists, blinded to the allocation group, assessed the brain damage using a light microscope. The mHDS included three alterations: SEND, pyknosis, and edema. Regions assessed were the Neocortex, Hippocampus, Thalamus, and Cerebellum. In each region, a score ranging from 0 to 4 was assigned based on the severity of the injury. SEND and pyknosis (both weighting factor 2) were scored based on percentage of damaged cells: 0% (0 p), 1–30% (1 p), 31–60% (2 p), 61–90% (3 p), and > 90% (4 p). Edema (weighting factor 1) was judged as: not present (0 p), minimal (1 p), mild (2 p), moderate (3 p), or severe (4 p). The score for each injury type was multiplied by its corresponding weighting factor and summed to yield a total for each region. The sum of all four regions was divided by four to attain a final mHDS. Animals that did not survive until day 7 were given a supramaximal total score (1 p). Additionally, the hippocampal SEND score was reported separately, graded on a scale of 0–4.

A model for neurocognitive testing (NCT) was performed [26]. The time to locate and open a food trough within a set of three differently colored troughs was recorded (maximum 300 s) before and after the intervention. A neurologic deficit score (NDS) was evaluated before and after the intervention [27]. The scale ranged from 0 points (equivalent to no damage) to 400 points (equivalent to brain death). The investigators performing NCT and NDS testing were not blinded to allocation groups due to practical difficulties. For further details of NCT and NDS, see Appendix A1.

Plasma EDTA samples were collected at baseline and at 2, 12, 24 and 48 h after randomization, as well as on day 7. Samples were centrifuged (2000 G, 10 min) and stored at − 80 °C. Neurofilament light chain (NfL), tau protein, and glial fibrillary acidic protein (GFAP) were measured using a single-molecule array (Simoa) multiplex immunoassay (Quanterix, Billerica, MA) by investigators blinded to the allocation groups. Analyses were performed at the Clinical Neurochemistry Laboratory at the University of Gothenburg.

### Statistics

To account for the small sample size, we employed non-parametric tests with effect sizes and bootstrap confidence intervals, recognizing that p-values are based on asymptotic approximations and should therefore be interpreted with caution. Between-group differences in cooling times were quantified using the Hodges-Lehmann estimator with 95% confidence intervals, alongside Mann–Whitney U tests [28]. Statistical testing of outcomes was performed for time points after the intervention.

The histological outcomes, mHDS and SEND, were compared across groups using the Kruskal–Wallis test, with effect size reported as epsilon-squared (ε^2^). The epsilon-squared represents the proportion of variance in ranks attributable to group differences (0 = no effect, 1 = complete separation) [29]. Pairwise contrasts (EH vs NT; DH vs NT) are presented as Cliff’s delta (δ) with 95% confidence intervals (CIs) estimated with 5000 bootstrap resamples [30]. Analyses were also repeated in survivors only. Day 7 functional outcomes (NCT and NDS) were analyzed analogously (Kruskal–Wallis, Cliff’s δ). NfL concentrations were log-transformed and analyzed in a group × time factorial model with cluster-robust standard errors by animal. Overall group × time effects were assessed using a Wald test. Prespecified contrasts (EH vs NT; DH vs NT) at 48 h and day 7 are reported as geometric mean ratios (GMRs) with 95% CIs, adjusted for Holm’s method across the four comparisons [31, 32]. Pairwise Mann–Whitney U tests were also performed.

To further address multiple testing, we used a Bonferroni-style correction of the significance levels: 2.5% for double pairwise comparisons at a single time point or a single comparison at two time points, and 1.25% for double pairwise comparisons at two time points.

Correlations between outcomes were assessed with Spearman’s rank correlation. Continuous and ordinal data are presented as medians with interquartile ranges (25th and 75th percentiles); categorical data as counts. A post-hoc power analysis was performed to estimate the minimum detectable differences in mHDS and SEND at 80% power.

Statistical analyses were performed using R version 4.1.2 (R Core Team, 2021) and RStudio version 2023.03.0 + 386.

## Results

### Study population and survival

A total of 42 animals were enrolled in the study. One animal developed ventricular fibrillation before induction and did not achieve baseline conditions, and eight animals failed to achieve ROSC, yielding an overall ROSC rate of 80%. The remaining 33 animals (11 per group) were randomized. Of these, 23 survived to the end of the study: nine in the NT group, eight in the EH group, and six in the DH group. One EH animal experienced re-fibrillation after stable ROSC, which was successfully defibrillated within one minute. Nine animals were euthanized within 24 h of the end of intervention (31–51 h after inclusion) due to failure to wean or combined general and respiratory distress following extubation. One additional animal developed late-onset respiratory distress and was euthanized on day 5. A flowchart of enrolment and survival is provided in Appendix A2. The characteristics of the included animals are specified in Table 1*.* The physiological parameters, arterial blood gas values, and cumulative doses of infusions administered during intervention are presented in Appendix A3*.*

**Table 1:** Characteristics of included animals per group, presented as median [25%; 75%], except re-fibrillations (n). No-flow time = untreated ventricular fibrillation; Low-flow time = ongoing mechanical compressions and/or a systolic blood pressure below 60 mmHg; ROSC = Return of spontaneous circulation; CPR = cardiopulmonary resuscitation; ROSC interval = time from initial ROSC to stable ROSC

Parameter	Normothermia (n = 11)	Early hypothermia (n = 11)	Delayed hypothermia (n = 11)
Weight (kg)	61 [57;64]	59 [55;60.5]	60 [57.5;64]
Induction time (s)	11 [8;14.5]	8 [6.5;13]	13.5 [6.5;20]
No-flow time (min)	10 [10;10]	10 [10;10]	10 [10;10]
Low-flow time (min)	8 [6;8]	8 [6;9]	6 [6;7]
Time from cardiac arrest to initial ROSC (min)	16 [15;16]	16 [16;18]	16 [14;16]
Time from cardiac arrest to stable ROSC (min)	28 [26;30]	28 [26;31.5]	26 [26;27]
Mean arterial pressure during initial CPR (mmHg)	43 [40.5;54]	46 [40;50]	45 [40.5;48]
Number of shocks (n)	4 [3;5]	4 [3;5.5]	3 [2.5;4]
Accumulated adrenaline boluses (mg)	2 [2;2.5]	2 [2;3]	2 [2;2]
Accumulated amiodarone boluses (mg)	300 [300;375]	450 [300;450]	300 [300;300]
Re-fibrillations in ROSC interval (n)	3	1	1
Survival (days)	7 [7;7]	7 [4.5;7]	7 [2;7]

### Temperature kinetics

**Fig. 1:**
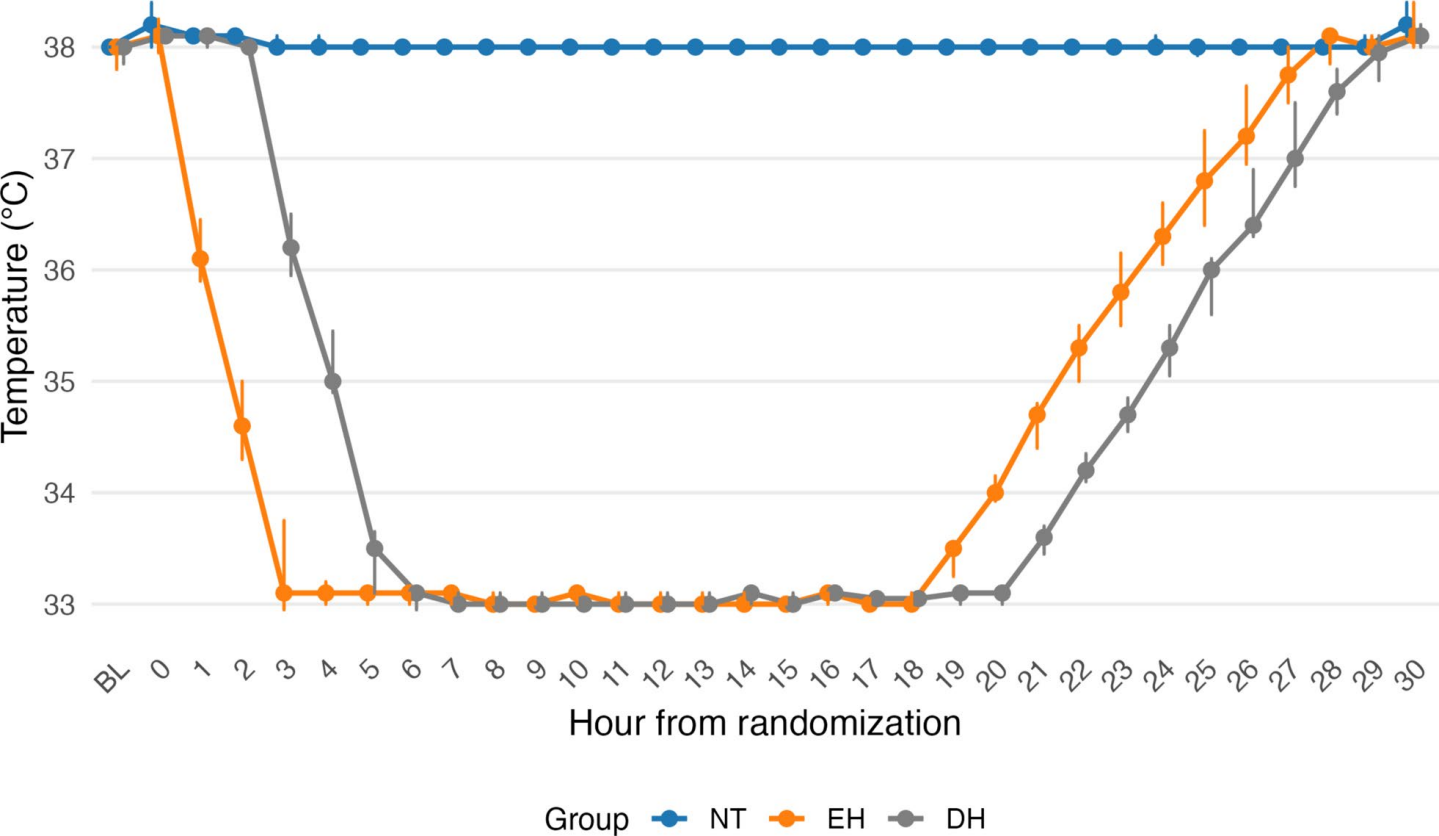
Temperatures during the intervention, medians with interquartile range. *BL* Baseline, *NT* Normothermia, *EH* Early hypothermia, *DH* Delayed hypothermia

The cooling trajectories are shown in Fig. 1. EH animals reached hypothermia significantly faster than DH animals: the median time to 34 °C was 145 min [125–186] in EH vs 282 min [267–304] in DH (*p* < 0.001; Hodges–Lehmann difference − 140 min [− 179 to − 96]). Similarly, the median time to 33.2 °C was 169 min [157–225] in EH vs 321 min [296–345] in DH (*p* < 0.001; Hodges–Lehmann difference − 145 min [− 180 to − 85]). Once the target temperature was reached, the median times at target temperature (33.2 °C) in the EH group were 947 [877–985] minutes and 915 [887–934] minutes in the DH group (*p* = 0.0014, Hodges–Lehmann difference + 40 min [− 48 to + 90]).

### Primary outcome

**Fig. 2:**
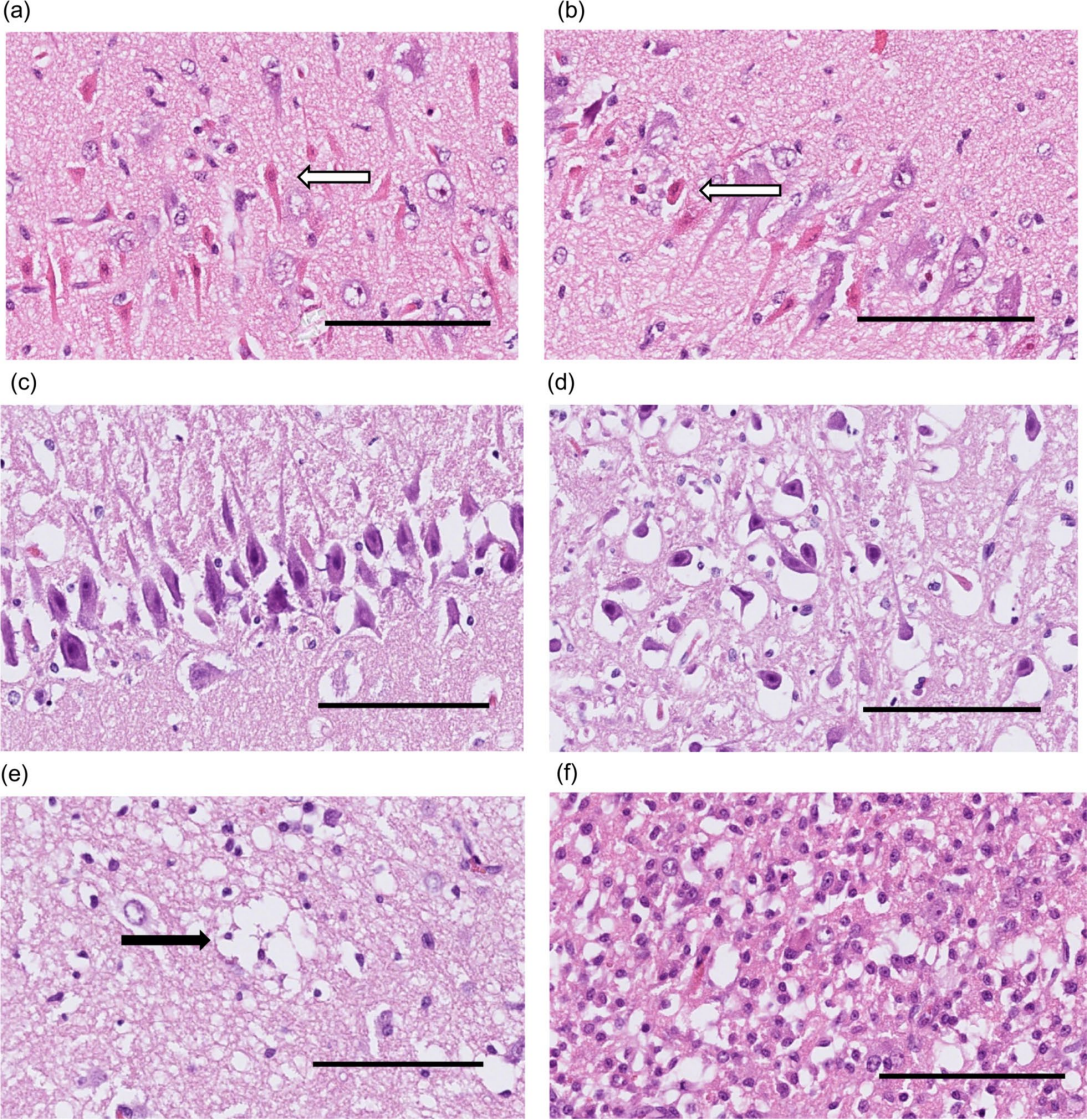
Microphotographs of histopathologic injuries, hematoxylin–eosin staining, Bar = 100 µm. **a** Selective eosinophilic neuronal death (SEND) in the hippocampus CA1 region; SEND (arrow) is seen among better-preserved neurons. **b** SEND (arrow) with marked shrinkage among better preserved neurons. **c** Pyknotic neurons (all cells) in the hippocampus CA1 region. **d** Pyknotic cells with marked shrinkage and perineuronal vacuoles (all cells) in the hippocampus, CA4 region. **e** Marked edema with clustered vacuoles (arrow) and shrunken cell nuclei. **f** Recent infarction with reactive cells and edema

The histopathological injuries are exemplified in Fig. 2. SEND and pyknosis could not be simultaneously detected in neurons. Our maximum mHDS was therefore adjusted from 20 to 14, as shown in Appendix A4.

**Fig. 3:**
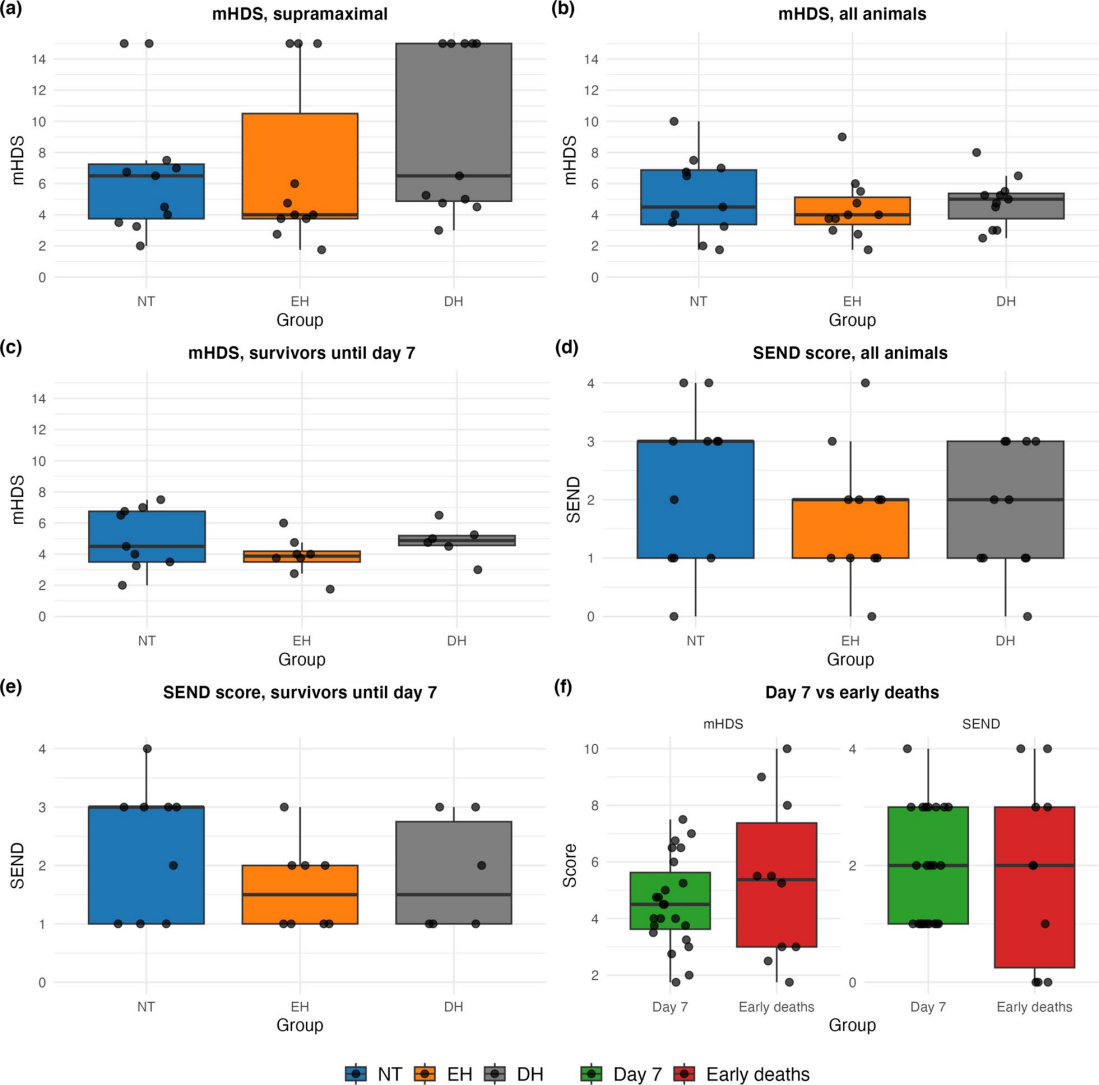
Histopathological results. Boxplots and all values of: **a** mHDS in all animals with supramaximal scores for early deaths (n = 33). **b** mHDS in all animals without supramaximal scores for early deaths (n = 33). **c** mHDS in survivors until day 7 (n = 23). **d** SEND scores in the hippocampus in all animals (n = 33). **e** SEND scores in the hippocampus in survivors until day 7 (n = 23). **f** mHDS in survivors until day 7 vs early deaths and SEND scores in the hippocampus in survivors until day 7 vs early deaths. *mHDS* modified Histology Damage Score, *SEND* Selective Eosinophilic Neuronal Death, *NT* normothermia, *EH* early hypothermia, *DH* delayed hypothermia

The mHDS scores are displayed in Fig. 3a-c. In all animals, including supramaximal scores, median values were 6.5 [3.8–7.3] for NT, 4.0 [3.8–10.5] for EH, and 6.5 [4.9–15.0] for DH (Kruskal–Wallis *p* = 0.29, ε^2^ = 0.078). Pairwise Cliff’s δ showed small, non-significant effects: EH vs NT δ = − 0.18 (− 0.65 to 0.32); DH vs NT δ = − 0.04 (− 0.55 to 0.48). Excluding supramaximal scores for early deaths, the medians were 4.5 [3.4–6.9] for NT, 4.0 [3.4–5.1] for EH, and 5.0 [3.8–5.4] for DH (Kruskal–Wallis *p* = 0.67, ε^2^ = 0.025) for all animals. Pairwise Cliff’s δ again indicated negligible effects: EH vs NT δ = − 0.33 (− 0.81 to 0.24); DH vs NT δ = − 0.04 (− 0.63 to 0.57). Among survivors until day 7, median scores were 4.5 [3.5–6.8] for NT, 3.9 [3.5–4.2] for EH, and 4.9 [4.6–5.2] for DH (Kruskal–Wallis *p* = 0.28, ε^2^ = 0.51). Pairwise Cliff’s δ yielded small, non-significant effects: EH vs NT δ = − 0.11 (− 0.58 to 0.39); DH vs NT δ = 0.26 (− 0.25 to 0.69). Post-hoc power analysis indicated that, with n = 11 per group and a pooled SD of ≈2.6, only between-group differences of ~ 3.3–3.5 mHDS scores or greater could be reliably detected with 80% power.

SEND scores are presented in Fig. 3d–e. In all animals, median SEND scores were 3 [1–3] for NT**,** 2 [1–2] for EH, and 2 [1–3]for DH (Kruskal–Wallis *p* = 0.53, ε^2^ = 0.040). Pairwise Cliff’s δ revealed small insignificant effects: EH vs NT δ = − 0.25 (− 0.70 to 0.23); DH vs NT δ = − 0.21 (− 0.64 to 0.26). Among survivors until day 7, medians were 3 [1–3] for NT, 1.5 [1–2] for EH, and 1.5 [1–2.8] for DH** (**Kruskal–Wallis *p* = 0.36, ε^2^ = 0). Pairwise Cliff’s δ again with negligible effects: EH vs NT δ = − 0.38 (− 0.82 to 0.17), DH vs NT δ = − 0.26 (− 0.76 to 0.30). Given the observed sample sizes (n = 11 per group) and variability (pooled SD ≈1.2), the post-hoc analysis indicated that only between-group differences of ~ 1.4–1.6 SEND scores or greater could be detected with 80% power.

As illustrated in Fig. 3f, the damage patterns of mHDS and SEND were similar when comparing survivors to early deaths. Baseline characteristics of survivors and non-survivors are shown in Appendix A5.

### Secondary outcomes

**Fig. 4:**
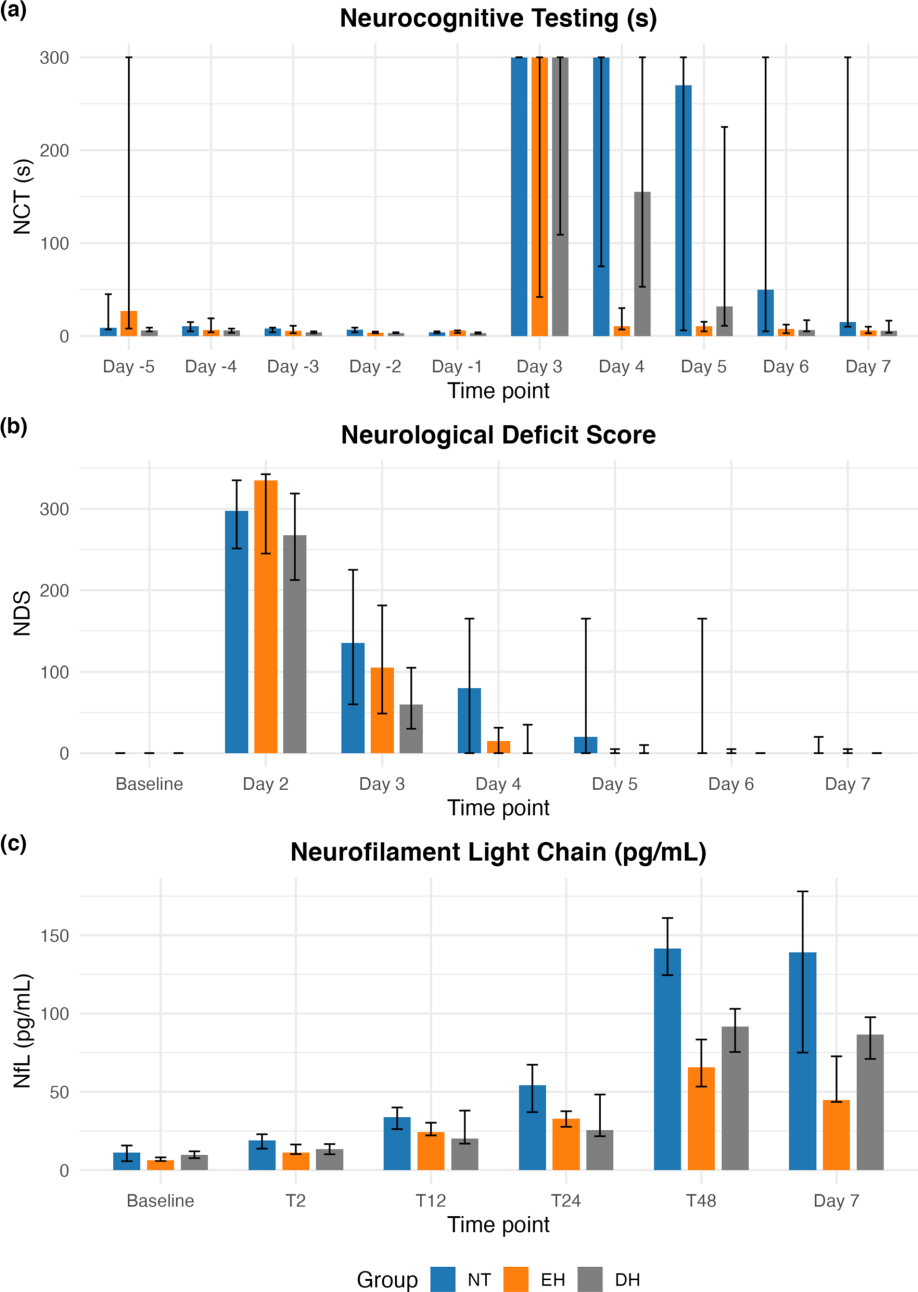
Secondary outcomes. **a** Neurocognitive testing (NCT) times (s); *p* = 0.11 on Day 7 (Kruskal–Wallis test). **b** Neurologic deficit scores (NDS); *p* = 0.67 on Day 7 (Kruskal–Wallis test). **c** Neurofilament light chain (NfL) levels (pg/mL): *p* = 0.0087 and *p* = 0.012 (Mann–Whitney U test) in early hypothermia (EH) vs normothermia (NT) at 48 h and on Day 7, respectively; and *p* = 0.075 and *p* = 0.33 (Mann–Whitney U test) in delayed hypothermia (DH) vs NT at the same time points. All medians with interquartile range. Time points T2–T48 representing hours from randomization

NCT times are presented in Fig. 4a and Appendix A6. Pre-interventional testing demonstrated similar learning patterns across the groups. On day 7, the median NCT times were 15 s [10–300] in the NT group, 6 s [3–10] in the EH group, and 5.5 s [3.5–16.5] in the DH group. Between-group comparison at day 7 showed small non-significant effects (Kruskal–Wallis *p* = 0.11, ε^2^ = 0.21) and pairwise Cliff’s δ: EH vs NT δ = − 0.60 (− 0.96 to − 0.11), DH vs NT δ = − 0.43 (− 0.93 to 0.19).

NDS results are shown in Fig. 4b and Appendix A7. Baseline testing was comparable across groups. On day 7, the median NDS values were 0 [0–20] in the NT group, 0 [0–5] in the EH group, and 0 [0–0] in the DH group. Group comparison confirmed small insignificant effects (Kruskal–Wallis *p* = 0.67, ε^2^ = 0.037) and pairwise Cliff’s δ: EH vs NT δ = − 0.13 (− 0.56 to 0.31), DH vs NT δ = − 0.20 (− 0.57 to 0.22).

Plasma biomarkers are shown in Fig. 4c and Appendix A8. GFAP and tau could not be measured due to a sequence mismatch with the human assays. Using a group × time model with cluster-robust standard errors, the overall interaction was significant (Wald χ^2^ = 12.4, *p* = 0.033). At 48 h, EH showed lower NfL than NT with a GMR of 0.51 (0.35–0.75; Holm-adjusted *p* = 0.0041). DH did not differ from NT: GMR = 0.71 (0.48–1.05; Holm-adjusted *p* = 0.081). At day 7, EH also showed a lower NfL than NT: GMR = 0.44 (0.27–0.71; Holm-adjusted *p* = 0.0054). DH did not differ from NT: GMR = 0.69 (0.42–1.14; Holm-adjusted *p* = 0.135). For completeness, descriptive medians were lower in EH vs NT at both 48 h (66 [53–83] vs 142 [125–161] pg/mL) and day 7 (45 [44–73] vs 139 [75–178] pg/mL), and Mann–Whitney comparisons yielded significant differences (*p* = 0.0087, *p* = 0.012). No significant differences were found in comparison of DH to NT at the same time points (*p* = 0.075 and *p* = 0.33). A worst-case sensitivity analysis (assigning non-survivors maximum + 1 pg/mL for Mann–Whitney testing) confirmed the EH vs NT difference at 48 h (*p* = 0.0076) but not at day 7 (*p* = 0.42). Comparisons of DH vs NT remained non-significant (48 h *p* = 0.11; day 7 *p* = 0.64). One extreme outlier (726 pg/mL at day 7 in an EH animal) was excluded; all other values for that animal were within the expected range.

### Correlation of outcomes

The results of the Spearman correlation analyses among survivors until day 7 are presented in Appendix A9. We observed a moderate correlation between mHDS and SEND (ρ≈0.54, *p* = 0.0078) and between NCT and NDS (ρ≈0.59, *p* = 0.0029). NfL at day 7 showed a strong correlation with NDS at day 7 (ρ≈0.65, *p* < 0.001) and a moderate correlation with NCT at day 7 (ρ≈0.49, *p* = 0.021). NfL levels at 48 h were strongly correlated with NfL at day 7 (ρ≈0.71, *p* < 0.001). No other outcome pairs demonstrated significant associations.

## Discussion

Our study showed no significant improvement in brain histopathology or in functional outcomes of induced hypothermia, delivered immediately after ROSC or with a two-hour delay, in young adult swine after cardiac arrest. By contrast, NfL was significantly reduced with immediate hypothermia at 48 h and Day 7.

In the present study, the time to reach 34 °C was just over 2 h using pre-inserted intravenous cooling catheters in paralyzed, human-sized animals. When cooling was delayed by 2 h, 34 °C was reached 4.5 h post-ROSC—which is faster than in large clinical trials [12, 13], especially considering the greater temperature delta due to a higher baseline temperature in swine. Animals were controlled at 38 °C before cooling, compared to a common admission temperature of 35–36 °C in humans [33]. Interestingly, increased temperatures were seen at inclusion, which justified a strategy of controlled normothermia as control.

Our primary outcome measure, mHDS [25], was aimed at addressing effects of attrition bias, with supramaximal scores for early deaths, based on the assumption that early death would be related to severity of the brain injury. Interestingly, the histopathological brain injury in early deaths compared to survivors until day 7 did not differ significantly, and the early deaths may thus have been related to factors other than the brain injury, such as respiratory failure. The underlying cause for the respiratory and general distress prompting preemptive euthanasia remains uncertain. Moreover, the characteristics of the animals surviving until day 7 compared to those dying early do not indicate any differences at inclusion.

The histopathological results of this study also indicate that the ischemic brain injury may be fully developed at 30–50 h post-ROSC, a time when most premature deaths occurred. A complicating factor, however, is the decomposition and reabsorption of damaged neurons over time, a process that has been described but remains poorly defined [34, 35]. Overall, no significant histopathological improvement was observed with either immediate or delayed induced hypothermia, regardless of assessment strategy.

Neither the NCT nor the NDS results demonstrate significant differences at the final follow-up on day 7. The recovery patterns for both outcomes, however, showed a trend towards quicker recovery in both hypothermia groups, particularly in the EH group. Previous studies have generally employed shorter follow-up periods [8, 9], and our results suggest that longer assessment periods may be necessary for the recovery of functional outcomes in large animals.

Interestingly, the NfL results showed that immediately induced cooling (EH) lowered NfL in plasma compared to normothermia (NT), whereas a delay of hypothermia by two hours (DH) abolished this effect. These findings are largely consistent with our preliminary results presented in a conference abstract [36]. The NfL protein is a subunit of neurofilaments, structural proteins found in the neuronal cytoplasm, particularly in large myelinated axons [37]. NfL has shown excellent predictive ability after cardiac arrest in humans [38]. The explanation for the discrepancy between outcomes (brain histopathology and functional outcomes versus NfL) is unclear. Still, NfL levels in plasma may be a more sensitive indicator of neuronal injury than other, coarser measures.

Our overall findings may suggest that a more severe brain injury is required; this should be addressed in future large animal experiments. In addition, future studies could benefit from the use of composite outcome measures integrating histology, neurocognitive tests, and biomarkers.

Our results demonstrate that early cooling is necessary to have an impact on NfL plasma levels in large mammals and may indicate a neuroprotective effect when the intervention is induced immediately after ROSC and under optimal conditions. Novel techniques for very early cooling have emerged, including liquid ventilation and intranasal cooling devices, and their feasibility and efficacy will be further explored [39, 40].

### Strengths and limitations

The strengths of this study include a robust design in accordance with updated guidelines, a long follow-up period, and homogeneity between groups at inclusion and throughout the intervention. The size of the investigated animals is comparable to that of human adults, which strengthens translational relevance. The study can also be considered large in an experimental context, but our post-hoc analysis suggests that it may have been underpowered to detect differences in the primary outcome.

A major limitation is the number of early deaths. The early drop-outs, combined with a statistical ceiling effect due to scale reduction, may have negatively impacted the discriminatory power.

Although neuron-specific enolase (NSE) was included in the preregistered protocol as a planned outcome, it could not be measured due to assay unavailability, which is a limitation.

The almost complete recovery of functional outcomes and lower-than-estimated mHDS scores indicate a milder brain injury than anticipated. Longer no-flow times may be needed in future studies to detect an intervention effect, but would yield lower ROSC rates. Furthermore, the substantial variability within the groups may suggest that a larger sample size is required.

## Conclusions

This study did not demonstrate a beneficial effect of hypothermia, whether initiated immediately or two hours after ROSC, compared with normothermia on histological or functional outcomes in adult-sized swine. However, immediately induced hypothermia was associated with lower NfL levels at both 48 h and 7 days, suggesting a potential mitigation of neuronal injury.

## Supplementary Information

Supplementary Material 1.

## Data Availability

The datasets generated and analyzed during this study are available upon request from the corresponding author.

## Abbreviations

CI: Confidence interval
DH: Delayed hypothermia
EH: Early hypothermia
GFAP: Glial fibrillary acidic protein
GMR: Geometric mean ratio
mHDS: Modified Histology damage score
NCT: Neurocognitive testing
NDS: Neurologic deficit score
NfL: Neurofilament light chain
NSE: Neuron-specific enolase
NT: Normothermia
OHCA: Out-of-hospital cardiac arrest
ROSC: Return of spontaneous circulation
SEND: Selective eosinophilic neuronal death
Simoa: Single molecule array
VF: Ventricular fibrillation
