# Supplementary material for: Hypothermia after Cardiac Arrest in Large Animals (HACA-LA): a randomized controlled experimental study
Source: Intensive Care Med Exp. 2025 Oct 22;13:105. doi: 10.1186/s40635-025-00815-y (PMC12545941; doi:10.1186/s40635-025-00815-y)
Supplement: Supplementary file 1 — Supplementary Material 1. [file 40635_2025_815_MOESM1_ESM.docx]

Appendix A

Hypothermia After Cardiac Arrest in Large Animals (HACA-LA): A Randomized Controlled Experimental Study

**A1. Neurocognitive testing and Neurologic deficit score**

**Figure A1**. Neurocognitive testing (NCT) **(a)** and Neurologic deficit score (NDS) time points **(b)**

**(a)**


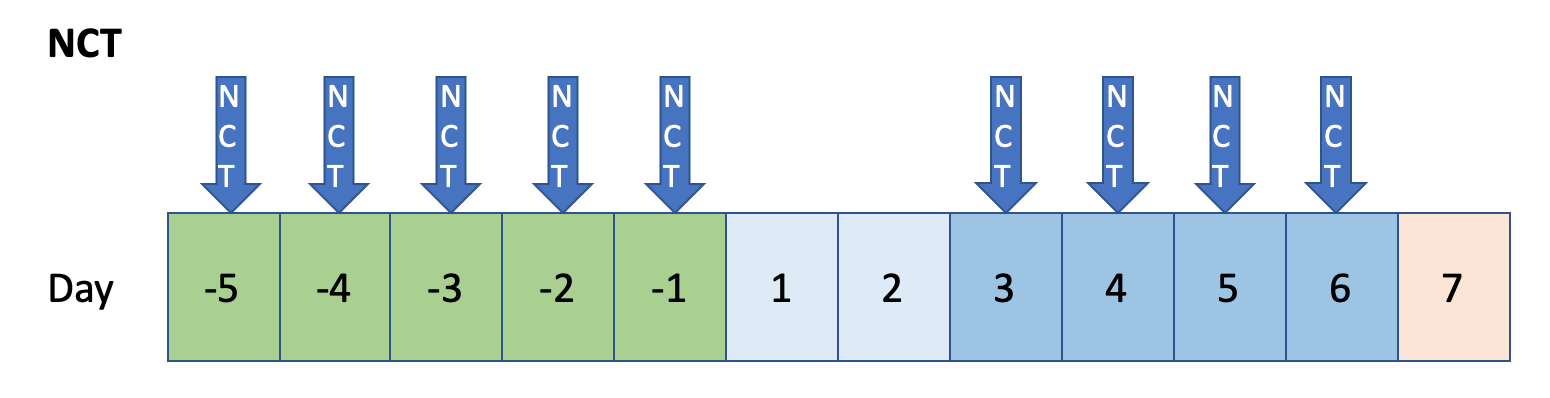


**(b)**


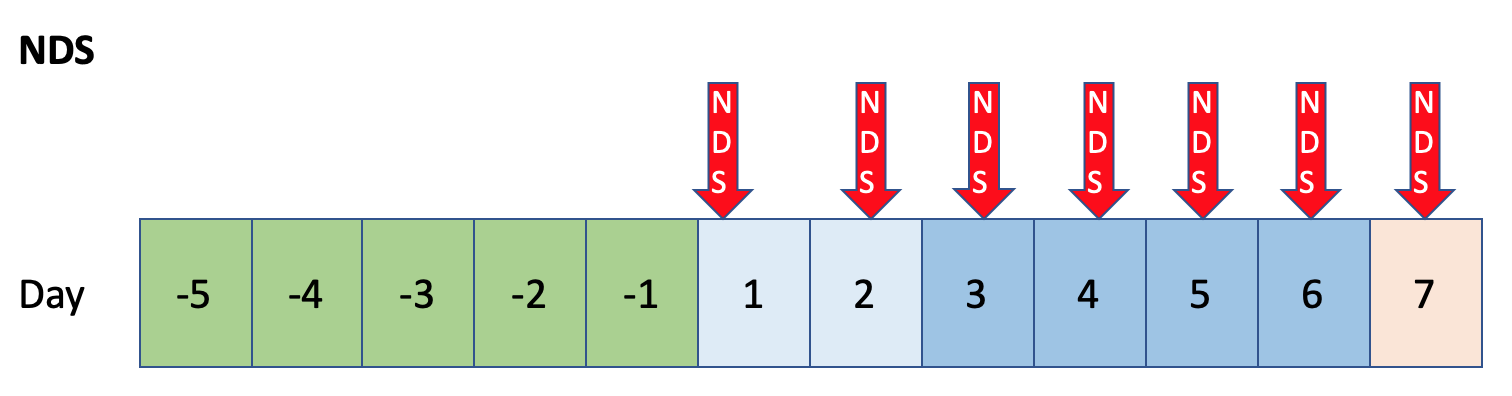


**Table A1.** Neurologic deficit score (NDS) protocol (from Sipos et al. 2008).

| **Examination and score (0 = normal)** | **Instruction** |
| --- | --- |
| **MENTAL STATUS**  0 = normal  50 = not normal nor comatose  100 = comatose | 0 = normal: pig specific behaviour (highly interested in the environment, showing this by walking around, trying to examine everything with the snout and giving characteristic grunting sounds)  50 = not normal nor comatose  100 = comatose. Lateral position, possibly running movements, no reaction to any stimuli |
| **BREATHING**  0 = normal; 10 = abnormal; 20 = apnea | Abnormal: irregular, tachypnoea (>30/min), bradypnoe (<18/min) |
| **CRANIAL NERVES** |  |
| Pupillary light reflex: Right: 0/5; Left 0/5 | Shining a light in each eye and observing for pupillary constriction |
| Stabismus: Right: 0/5; Left 0/5 | Fixed deviation of globes |
| Nystagmus: Right: 0/5; Left 0/5 | Spontaneous nystagmus |
| Corneal reflex: Right: 0/5; Left 0/5 | Blink response to tactile stimulus of the cornea with a cotton swab |
| Ear reflex: Right: 0/5; Left 0/5 | Finger introduced into outer auditory canal, followed by twitch of the auricle |
| Menace reaction: 0/20 | Making a sudden, threatening gesture with the hand in the direction of one eye |
| Snout reflex: 0/20 | Pinching the nasal septum with two fingers |
| Swallowing reflex: 0/20 | Some water is to be administered laterally into the mouth with syringe |
| **MOTOR AND SENSORIC FUNCTION** |  |
| Muscle tonus: Foreleg 0/5; Hind leg 0/5 | Normal = neither spasticity nor paresis/paralysis |
| Positioning reaction: Foreleg 0/5; Hind leg 0/5 | Distal extremity is flexed, the pig should immediately return the foot to a normal position |
| Flexor reflex: Foreleg 0/5; Hind leg 0/5 | Flexor/withdrawal reflex, after pricking with a needle to interdigital cleft |
| Hopping reaction: Foreleg 0/15; Hind leg 0/15 | Shifting the animal to one side. First tries to oppose lateral pressure, then hops on the contralateral limb |
| Dorsal pressure reaction: Foreleg 0/15; Hind leg 0/15 | Pressure to the back is responded by contra-pressure |
| Panniculus reflex: 0/10 | Smooth paravertebral prickling of the skin with a needle |
| Perineal reflex: 0/10 | Smoothly prickling the perineum with a needle. Response is a contraction of the anal sphincter muscle and a ventral flexion of the tail |
| Standing: 0/15/30 | 0= self, 15 = with help, 30 = not able |
| Gait: 0/15/30 | 0= self, 15 = with help, 30 = not able |
| **Total:** | 0–400 points |

**A2. Inclusion and survival flowchart**

**Figure A2**. Flowchart of inclusion and survival until day 7

**
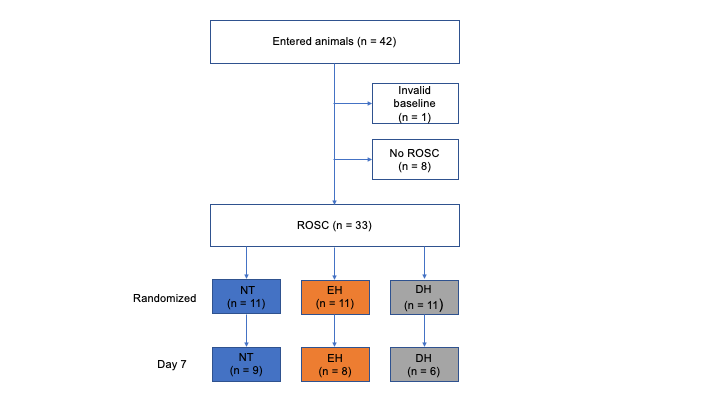
**

ROSC = Return of spontaneous circulation; NT = Normothermia; EH = Early hypothermia; DH = Delayed hypothermia.

**A3. Physiological parameters, arterial blood gas values and cumulative doses of infusions during intervention**

**Figure A3a.** The physiological parameters and arterial blood gas values are presented as median with interquartile range per group


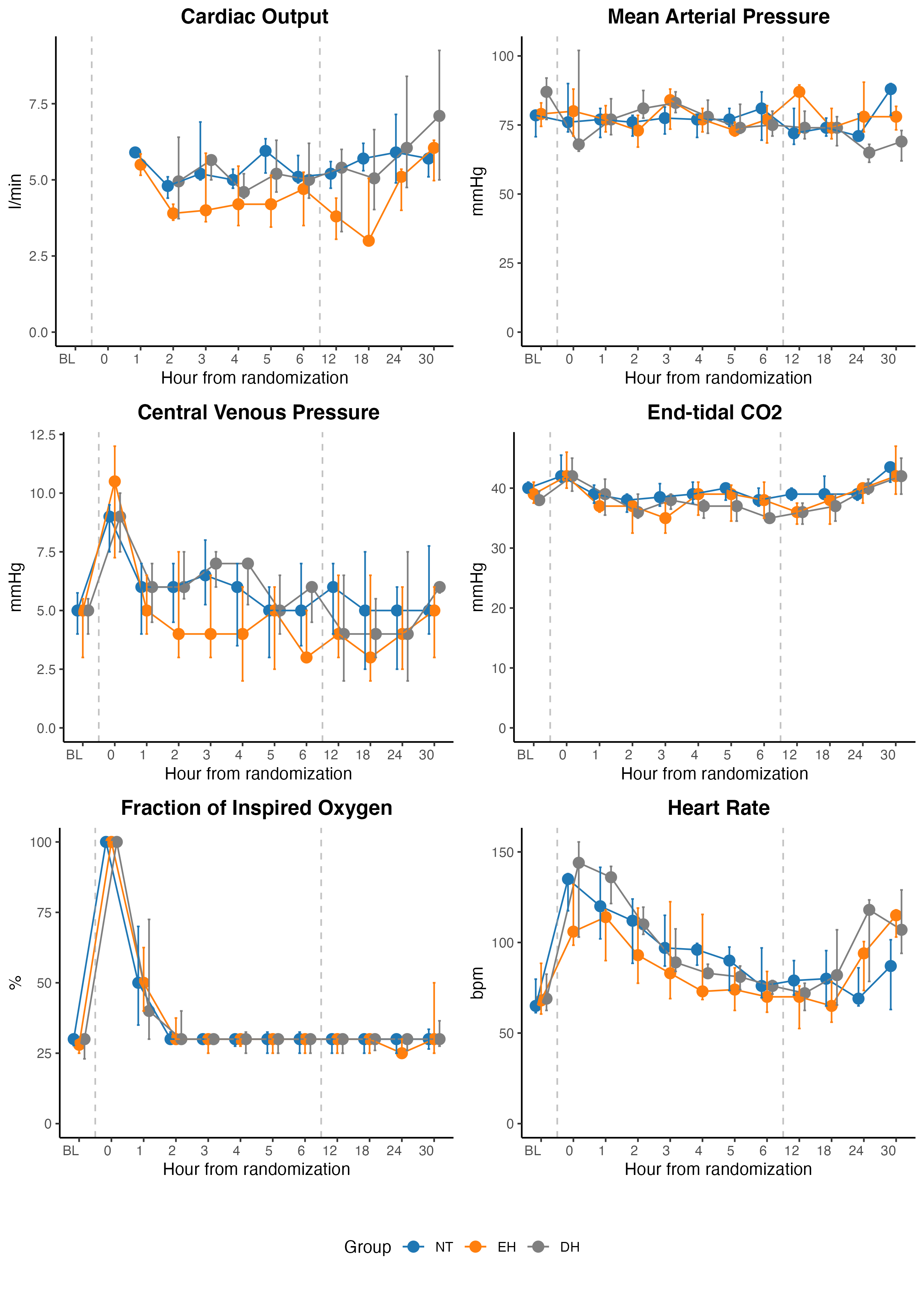


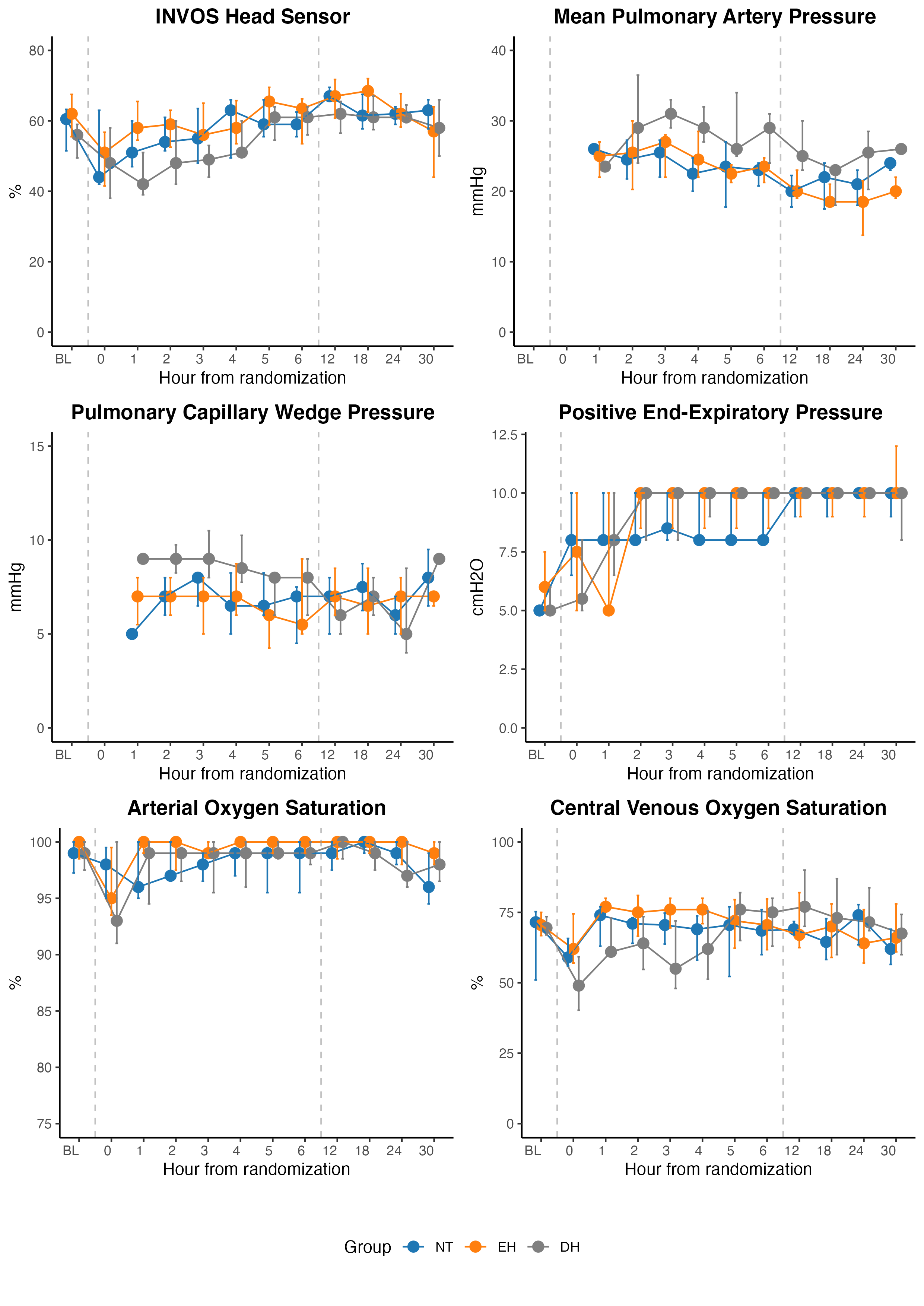


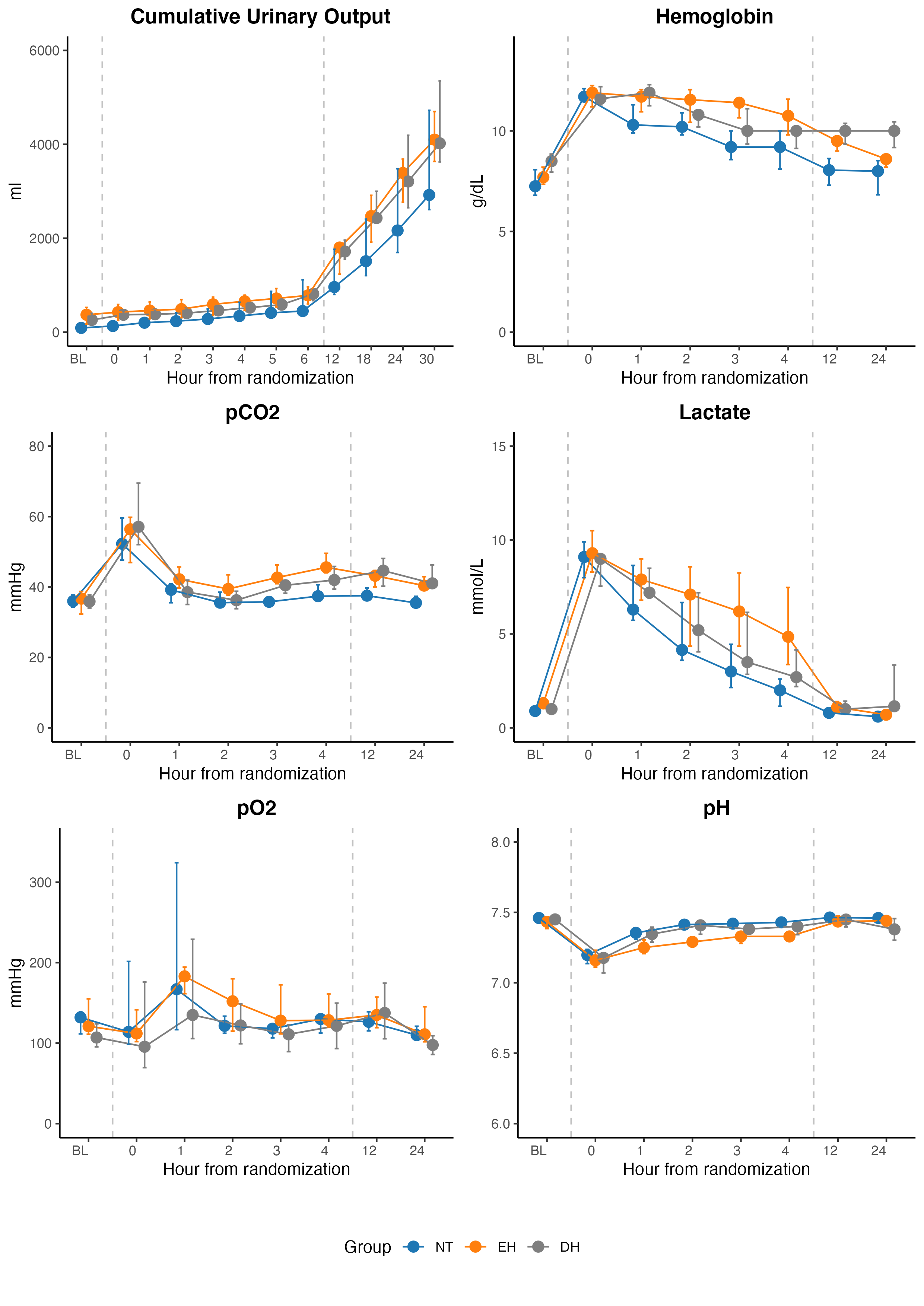
BL = Baseline; NT = Normothermia; EH = Early hypothermia; DH = Delayed hypothermia

**Figure A3b.** Cumulative doses of Infusions are presented as median with interquartile range per group


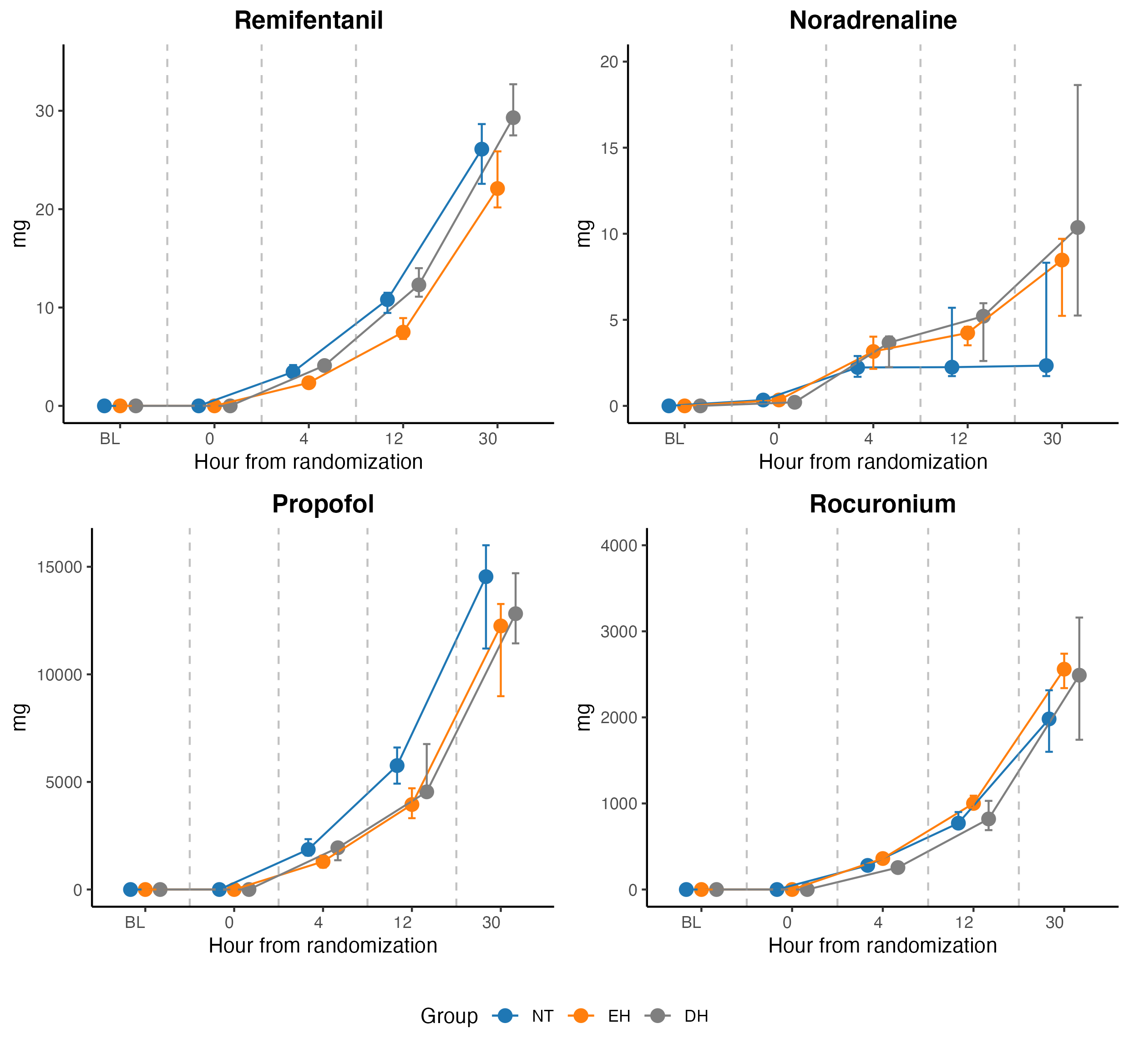


BL = Baseline; NT = Normothermia; EH = Early hypothermia; DH = Delayed hypothermia

**A4. mHDS scoring adjustment**

Our histological assessments of brain tissue showed that SEND and pyknosis could not both be detected in the same neurons, as displayed in Figure A4a-b. The pathological variables were thus mutually exclusive; that is, 100% presence of SEND meant 0% presence of pyknosis, rendering 4 p and 0 p for SEND and pyknosis respectively – or vice versa.

The scoring of SEND and pyknosis was based on a predefined percentage of damaged cells: 0% (0 p), 1–30% (1 p), 31–60% (2 p), 61–90% (3 p) and >90% (4 p) of each type of cell damage. Consequently, a >90% presence of SEND (rendering 4 p) still allowed 1-9% presence of pyknosis (rendering 1 p) totaling a maximum 5 p for both injuries.

The mutually exclusive damage patterns with only partial overlap meant that the maximal score for SEND and pyknosis combined was 10 when multiplied by the corresponding weighting factor (2 for both). The original predefined score was based on parallel presence of SEND and pyknosis with a maximal score of 16 for both. The overall maximal mHDS score was hence adjusted from 20 to 14, and animals not surviving until day 7 were given a supramaximal mHDS score of 15 instead of 21.

**Figure A4a-b.** Microphotographs of SEND (white arrowhead) and pyknosis (black arrowhead) concurring in close proximity, hematoxylin-eosin staining. Bar = 100 micrometers.


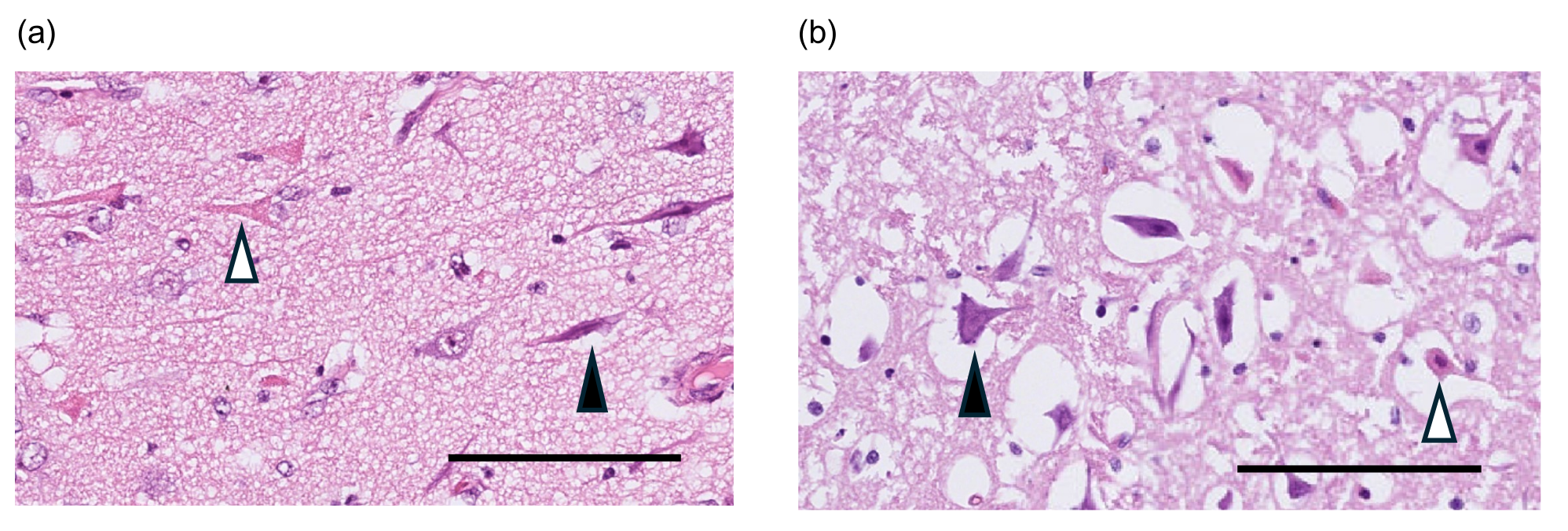


**A5. Characteristics of survivors until day 7 and early deaths**

**Table A5.** Characteristics of survivors until day 7 and early deaths are presented as median [25%;75%], except re-fibrillations, presented as numbers

| Parameter | Survivors until day 7 | Early deaths |
| --- | --- | --- |
| Weight (kg) | 60 [57;62.5] | 57.5 [56;63.5] |
| Induction time (s) | 10 [7;16] | 9 [6;21] |
| No-flow time (min) | 10 [10;10] | 10 [10;10] |
| Low-flow time (min) | 6 [6;8] | 8 [6;9] |
| Time from cardiac arrest to initial ROSC (min) | 16 [14;17] | 16 [16;17.5] |
| Time from cardiac arrest to stable ROSC (min) | 26 [26;29] | 29 [26;31] |
| Mean arterial pressure during initial CPR (mmHg) | 43 [39;48] | 48 [41;51] |
| Number of shocks (n) | 3 [3;4.5] | 4.5 [3;7.5] |
| Accumulated adrenaline bouses (mg) | 2 [2;2.5] | 2 [2;3] |
| Accumulated amiodarone boluses (mg) | 300 [300;450] | 300 [300;450] |
| Re-fibrillations in ROSC interval (n) | 4 | 1 |

No-flow time = untreated ventricular fibrillation; Low-flow time = ongoing mechanical compressions and/or a systolic blood pressure below 60 mmHg; ROSC = Return of spontaneous circulation; CPR = cardiopulmonary resuscitation; ROSC interval = time from initial ROSC to stable ROSC.

**A6. Neurocognitive testing results**

**Table A6.** Neurocognitive testing (NCT) results, median (25%, 75%) (s)

| **Time point** | **Statistic** | **NT (n = 11)** | **EH (n = 11)** | **DH (n = 11)** | **Total (n = 33)** |
| --- | --- | --- | --- | --- | --- |
| Day -5 | Mean (SD) | 78.1 (126.5) | 139.7 (152.2) | 36.4 (88.2) | 81.4 (126.0) |
|  | Median (Min, Max) | 9.0 (3.0, 300.0) | 27.0 (5.0, 300.0) | 6.0 (3.0, 300.0) | 8.0 (3.0, 300.0) |
|  | MIssing (n, %) | 2 (18.2%) | 2 (18.2%) | 0 (0.0%) | 4 (12.1%) |
| Day -4 | Mean (SD) | 39.8 (91.8) | 39.0 (92.1) | 7.4 (5.4) | 28.0 (73.0) |
|  | Median (Min, Max) | 10.5 (3.0, 300.0) | 6.5 (3.0, 300.0) | 6.0 (3.0, 18.0) | 6.0 (3.0, 300.0) |
|  | MIssing (n, %) | 1 (9.1%) | 1 (9.1%) | 0 (0.0%) | 2 (6.1%) |
| Day -3 | Mean (SD) | 33.5 (88.5) | 7.4 (5.1) | 4.0 (1.5) | 15.2 (52.1) |
|  | Median (Min, Max) | 8.0 (3.0, 300.0) | 5.5 (3.0, 16.0) | 4.0 (1.0, 7.0) | 4.5 (1.0, 300.0) |
|  | MIssing (n, %) | 0 (0.0%) | 1 (9.1%) | 0 (0.0%) | 1 (3.0%) |
| Day -2 | Mean (SD) | 9.5 (9.4) | 5.0 (4.4) | 4.0 (2.3) | 6.2 (6.5) |
|  | Median (Min, Max) | 7.0 (3.0, 34.0) | 3.5 (2.0, 17.0) | 3.0 (2.0, 10.0) | 4.0 (2.0, 34.0) |
|  | MIssing (n, %) | 0 (0.0%) | 1 (9.1%) | 0 (0.0%) | 1 (3.0%) |
| Day -1 | Mean (SD) | 5.0 (4.8) | 7.5 (7.1) | 3.4 (1.1) | 5.2 (5.0) |
|  | Median (Min, Max) | 4.0 (1.0, 19.0) | 6.0 (1.0, 24.0) | 3.0 (2.0, 5.0) | 4.0 (1.0, 24.0) |
|  | MIssing (n, %) | 0 (0.0%) | 1 (9.1%) | 0 (0.0%) | 1 (3.0%) |
| Day 3 | Mean (SD) | 271.1 (77.7) | 199.8 (138.5) | 203.4 (127.0) | 227.6 (115.5) |
|  | Median (Min, Max) | 300.0 (65.0, 300.0) | 300.0 (22.0, 300.0) | 300.0 (6.0, 300.0) | 300.0 (6.0, 300.0) |
|  | MIssing (n, %) | 2 (18.2%) | 3 (27.3%) | 4 (36.4%) | 9 (27.3%) |
| Day 4 | Mean (SD) | 210.0 (136.4) | 52.0 (101.9) | 166.7 (135.1) | 144.7 (138.6) |
|  | Median (Min, Max) | 300.0 (7.0, 300.0) | 10.5 (3.0, 300.0) | 155.0 (6.0, 300.0) | 87.5 (3.0, 300.0) |
|  | MIssing (n, %) | 2 (18.2%) | 3 (27.3%) | 4 (36.4%) | 9 (27.3%) |
| Day 5 | Mean (SD) | 172.9 (145.3) | 45.6 (102.9) | 115.1 (136.0) | 113.6 (135.5) |
|  | Median (Min, Max) | 270.0 (5.0, 300.0) | 10.5 (3.0, 300.0) | 32.0 (2.0, 300.0) | 15.5 (2.0, 300.0) |
|  | MIssing (n, %) | 2 (18.2%) | 3 (27.3%) | 4 (36.4%) | 9 (27.3%) |
| Day 6 | Mean (SD) | 143.8 (148.9) | 43.9 (103.6) | 56.5 (119.5) | 86.3 (130.3) |
|  | Median (Min, Max) | 50.0 (4.0, 300.0) | 7.5 (3.0, 300.0) | 6.5 (1.0, 300.0) | 10.0 (1.0, 300.0) |
|  | MIssing (n, %) | 2 (18.2%) | 3 (27.3%) | 5 (45.5%) | 10 (30.3%) |
| Day 7 | Mean (SD) | 138.3 (153.4) | 42.5 (104.1) | 56.0 (119.7) | 83.5 (131.6) |
|  | Median (Min, Max) | 15.0 (4.0, 300.0) | 6.0 (2.0, 300.0) | 5.5 (2.0, 300.0) | 10.0 (2.0, 300.0) |
|  | MIssing (n, %) | 2 (18.2%) | 3 (27.3%) | 5 (45.5%) | 10 (30.3%) |

NT = Normothermia; EH = Early hypothermia; DH = Delayed hypothermia**.**

**A7. Neurologic deficit score results**

**Table A7.** Neurologic deficit score (NDS) results, median (25%, 75%)

| **Time point** | **Statistic** | **NT (n = 11)** | **EH (n = 11)** | **DH (n = 11)** | **Total (n = 33)** |
| --- | --- | --- | --- | --- | --- |
| Baseline | Mean (SD) | 0.0 (0.0) | 0.5 (1.5) | 0.0 (0.0) | 0.2 (0.9) |
|  | Median (Min, Max) | 0.0 (0.0, 0.0) | 0.0 (0.0, 5.0) | 0.0 (0.0, 0.0) | 0.0 (0.0, 5.0) |
|  | MIssing (n, %) | 0 (0.0%) | 0 (0.0%) | 0 (0.0%) | 0 (0.0%) |
| Day 2 | Mean (SD) | 289.4 (59.2) | 289.3 (88.9) | 262.5 (61.9) | 281.7 (68.7) |
|  | Median (Min, Max) | 297.5 (205.0, 360.0) | 335.0 (145.0, 370.0) | 267.5 (190.0, 320.0) | 310.0 (145.0, 370.0) |
|  | MIssing (n, %) | 3 (27.3%) | 4 (36.4%) | 5 (45.5%) | 12 (36.4%) |
| Day 3 | Mean (SD) | 137.8 (94.8) | 126.2 (116.2) | 77.9 (75.8) | 116.5 (97.0) |
|  | Median (Min, Max) | 135.0 (0.0, 235.0) | 105.0 (0.0, 340.0) | 60.0 (0.0, 215.0) | 105.0 (0.0, 340.0) |
|  | MIssing (n, %) | 2 (18.2%) | 3 (27.3%) | 4 (36.4%) | 9 (27.3%) |
| Day 4 | Mean (SD) | 91.7 (91.7) | 35.6 (63.9) | 50.7 (105.0) | 61.0 (87.6) |
|  | Median (Min, Max) | 80.0 (0.0, 225.0) | 15.0 (0.0, 190.0) | 0.0 (0.0, 285.0) | 20.0 (0.0, 285.0) |
|  | MIssing (n, %) | 2 (18.2%) | 3 (27.3%) | 4 (36.4%) | 9 (27.3%) |
| Day 5 | Mean (SD) | 83.9 (96.9) | 24.4 (61.3) | 52.9 (131.2) | 55.0 (97.8) |
|  | Median (Min, Max) | 20.0 (0.0, 220.0) | 0.0 (0.0, 175.0) | 0.0 (0.0, 350.0) | 0.0 (0.0, 350.0) |
|  | MIssing (n, %) | 2 (18.2%) | 3 (27.3%) | 4 (36.4%) | 9 (27.3%) |
| Day 6 | Mean (SD) | 65.6 (94.2) | 23.8 (59.5) | 3.3 (8.2) | 34.8 (71.2) |
|  | Median (Min, Max) | 0.0 (0.0, 205.0) | 0.0 (0.0, 170.0) | 0.0 (0.0, 20.0) | 0.0 (0.0, 205.0) |
|  | MIssing (n, %) | 2 (18.2%) | 3 (27.3%) | 5 (45.5%) | 10 (30.3%) |
| Day 7 | Mean (SD) | 41.7 (78.1) | 17.5 (42.0) | 3.3 (8.2) | 23.3 (55.3) |
|  | Median (Min, Max) | 0.0 (0.0, 200.0) | 0.0 (0.0, 120.0) | 0.0 (0.0, 20.0) | 0.0 (0.0, 200.0) |
|  | MIssing (n, %) | 2 (18.2%) | 3 (27.3%) | 5 (45.5%) | 10 (30.3%) |

NT = Normothermia; EH = Early hypothermia; DH = Delayed hypothermia.

**A8. NfL results**

**Table A8.** NfL results, median (25%, 75%) (pg/mL)

| **Time point** | **Statistic** | **NT (n = 11)** | **EH (n = 11)** | **DH (n = 11)** | **Total (n = 33)** |
| --- | --- | --- | --- | --- | --- |
| Baseline | Mean (SD) | 11.8 (8.2) | 7.5 (2.5) | 15.7 (15.4) | 11.5 (10.2) |
|  | Median (Min, Max) | 11.3 (3.9, 32.1) | 6.2 (5.5, 13.0) | 9.7 (6.4, 54.5) | 8.1 (3.9, 54.5) |
|  | MIssing (n, %) | 0 (0.0%) | 0 (0.0%) | 1 (9.1%) | 1 (3.0%) |
| T2 | Mean (SD) | 19.1 (7.8) | 14.6 (7.4) | 18.0 (17.3) | 17.2 (11.3) |
|  | Median (Min, Max) | 18.9 (8.5, 35.1) | 11.3 (8.6, 34.3) | 13.3 (5.3, 65.8) | 14.7 (5.3, 65.8) |
|  | MIssing (n, %) | 0 (0.0%) | 0 (0.0%) | 1 (9.1%) | 1 (3.0%) |
| T12 | Mean (SD) | 36.0 (18.1) | 26.7 (11.2) | 28.9 (17.1) | 30.6 (15.7) |
|  | Median (Min, Max) | 33.7 (12.4, 80.1) | 24.3 (12.8, 54.1) | 20.2 (9.3, 63.5) | 27.8 (9.3, 80.1) |
|  | MIssing (n, %) | 0 (0.0%) | 0 (0.0%) | 2 (18.2%) | 2 (6.1%) |
| T24 | Mean (SD) | 55.0 (24.9) | 41.2 (33.6) | 47.1 (46.4) | 47.8 (34.9) |
|  | Median (Min, Max) | 54.2 (18.5, 100.0) | 32.8 (17.1, 139.0) | 25.6 (19.9, 170.0) | 35.0 (17.1, 170.0) |
|  | MIssing (n, %) | 0 (0.0%) | 0 (0.0%) | 1 (9.1%) | 1 (3.0%) |
| T48 | Mean (SD) | 137.3 (28.5) | 77.6 (49.3) | 106.6 (61.2) | 105.0 (53.1) |
|  | Median (Min, Max) | 141.5 (79.5, 164.0) | 65.7 (32.3, 209.0) | 91.6 (54.5, 252.0) | 89.3 (32.3, 252.0) |
|  | MIssing (n, %) | 3 (27.3%) | 1 (9.1%) | 2 (18.2%) | 6 (18.2%) |
| Day 7 | Mean (SD) | 139.6 (71.5) | 56.8 (20.8) | 89.3 (32.4) | 99.6 (60.4) |
|  | Median (Min, Max) | 139.0 (60.5, 243.0) | 44.7 (35.2, 85.4) | 86.6 (50.5, 145.0) | 83.0 (35.2, 243.0) |
|  | MIssing (n, %) | 2 (18.2%) | 4 (36.4%) | 5 (45.5%) | 11 (33.3%) |

NT = Normothermia; EH = Early hypothermia; DH = Delayed hypothermia.

**A9. Correlation of outcomes**

**Table A9.** Correlations between outcomes in survivors until day 7

| **Outcomes** | **Spearman rho** | **p-value** |
| --- | --- | --- |
| mHDS vs SEND | 0.54 | 0.0078 |
| mHDS vs NCT | 0.32 | 0.14 |
| mHDS vs NDS | 0.064 | 0.77 |
| NCT vs SEND | 0.36 | 0.091 |
| NCT vs NDS | 0.59 | 0.0029 |
| NDS vs SEND | 0.0089 | 0.97 |
| NfL T48 vs mHDS | 0.14 | 0.53 |
| NfL T48 vs SEND | 0.27 | 0.22 |
| NfL T48 vs NCT | 0.31 | 0.16 |
| NfL T48 vs NDS | 0.39 | 0.072 |
| NfL T48 vs NfL D7 | 0.71 | <0.001 |
| NfL D7 vs mHDS | 0.38 | 0.076 |
| NfL D7 vs SEND | 0.14 | 0.54 |
| NfL D7 vs NCT | 0.49 | 0.021 |
| NfL D7 vs NDS | 0.65 | <0.001 |

The strength of Spearman's rho coefficients was interpreted as follows: 0.00–0.19 indicated a very weak correlation, 0.20–0.39 a weak correlation, 0.40–0.59 a moderate correlation, 0.60–0.79 a strong correlation, and 0.80–1.00 a very strong correlation. mHDS = modified Histology Damage Score; SEND = Selective Eosinophilic; NCT = Neurocognitive testing; NDS = Neurologic deficit score; NfL T48 = NfL levels at 48 hours from randomization; NfL D7 = NfL levels on day 7
